# Supplementary material for: Biparental Inheritance and Instability of kDNA in Experimental Hybrids of Trypanosoma cruzi: A Proposal for a Mechanism
Source: Biology (Basel). 2025 Oct 11;14(10):1394. doi: 10.3390/biology14101394 (PMC12562267; doi:10.3390/biology14101394)
Supplement: Supplementary file 1 [file biology-14-01394-s001.zip › Table S1.pdf]

Table S1. Parental and hybrid *T. cruzi* lines analyzed, with estimated generations in culture and NCBI SRA accession numbers.

T. cruzi line[1]	Generation after hybridization (approximated)	SRA Accession
P1 (Parent)	70	SRR15686217
P2 (Parent)	70	SRR15686216
P1-800-200	800	SRR15686200
P1-800-201	800	SRR15686201
P2-800-198	800	SRR15686198
P2-800-199	800	SRR15686199
1C2	95	SRR15686205
2C1	95	SRR15686203
1D12	95	SRR15686204
1C2-800-214	800	SRR15686214
1C2-800-213	800	SRR15686213
1C2-800-212	800	SRR15686212
2C1-800-208	800	SRR15686208
2C1-800-206	800	SRR15686206
2C1-800-207	800	SRR15686207
1D12-800-211	800	SRR15686211
1D12-800-212	800	SRR15686210
1D12-800-209	800	SRR15686209

[1] Suffixes -800-xxx indicate independent clones derived from each hybrid lineage after ~800 generations of *in vitro* culture where -xxx indicates the three last number from NCBI SRA accession. Re-cloning was performed at the end of the microevolution experiment, prior to sequencing.
