# Supplementary material for: Biparental Inheritance and Instability of kDNA in Experimental Hybrids of Trypanosoma cruzi: A Proposal for a Mechanism
Source: Biology (Basel). 2025 Oct 11;14(10):1394. doi: 10.3390/biology14101394 (PMC12562267; doi:10.3390/biology14101394)
Supplement: Supplementary file 1 [file biology-14-01394-s001.zip › Table S2.pdf]

Table S2. Read depth, copy number and normalization factors for estimating kDNA quantities.

	P1	P2	P1-800-200	P1-800-201	P2-800-198	P2-800-199	1C2	1D12	2C1	1C2-800_212	1C2-800_213	1C2-800_214	2C1-800_206	2C1-800_207	2C1-800_208	1D12-800_209	1D12-800_210	1D12-800_211
Median read depth																		
Chr1 (124,001-613,000)	123.3	188.3	77.3	73.3	121.0	222.1	36.6	29.0	44.1	21.2	33.1	20.9	66.4	12.7	113.8	46.0	61.6	27.5
Chr18 (300,001-500,000)	117.6	183.2	74.5	70.7	117.8	214.6	45.8	30.5	55.4	21.0	32.1	19.9	64.5	14.6	109.4	45.6	61.0	27.5
Copy number																		
Chr1 (124,001-613,000)	2	2	2	2	2	2	3	3	3	3	3	3	3	3	3	3	3	3
Chr18 (300,000-500,000)	2	2	2	2	2	2	4	3	4	4	3	3	3	3	3	3	3	3
Normalization factor																		
Chr1/n	61.7	94.1	38.7	36.6	60.5	111.1	12.2	9.7	14.7	7.1	11.0	7.0	22.1	4.2	37.9	15.3	20.5	9.4
Chr18/n	58.8	91.6	37.2	35.3	58.9	107.3	11.4	10.2	13.9	7.0	10.7	6.6	21.5	4.9	36.5	15.2	20.3	9.2
Avg	60.2	92.9	38.0	36.0	59.7	109.2	11.8	9.9	14.3	7.0	10.9	6.8	21.8	4.6	37.2	15.3	20.4	9.3
Std dev	1.4	1.3	0.7	0.7	0.8	1.9	0.4	0.2	0.4	0.0	0.2	0.2	0.3	0.3	0.7	0.1	0.1	0.0
