# Supplementary figures and images for: Biparental Inheritance and Instability of kDNA in Experimental Hybrids of Trypanosoma cruzi: A Proposal for a Mechanism
Source: Biology (Basel). 2025 Oct 11;14(10):1394. doi: 10.3390/biology14101394 (PMC12562267; doi:10.3390/biology14101394)

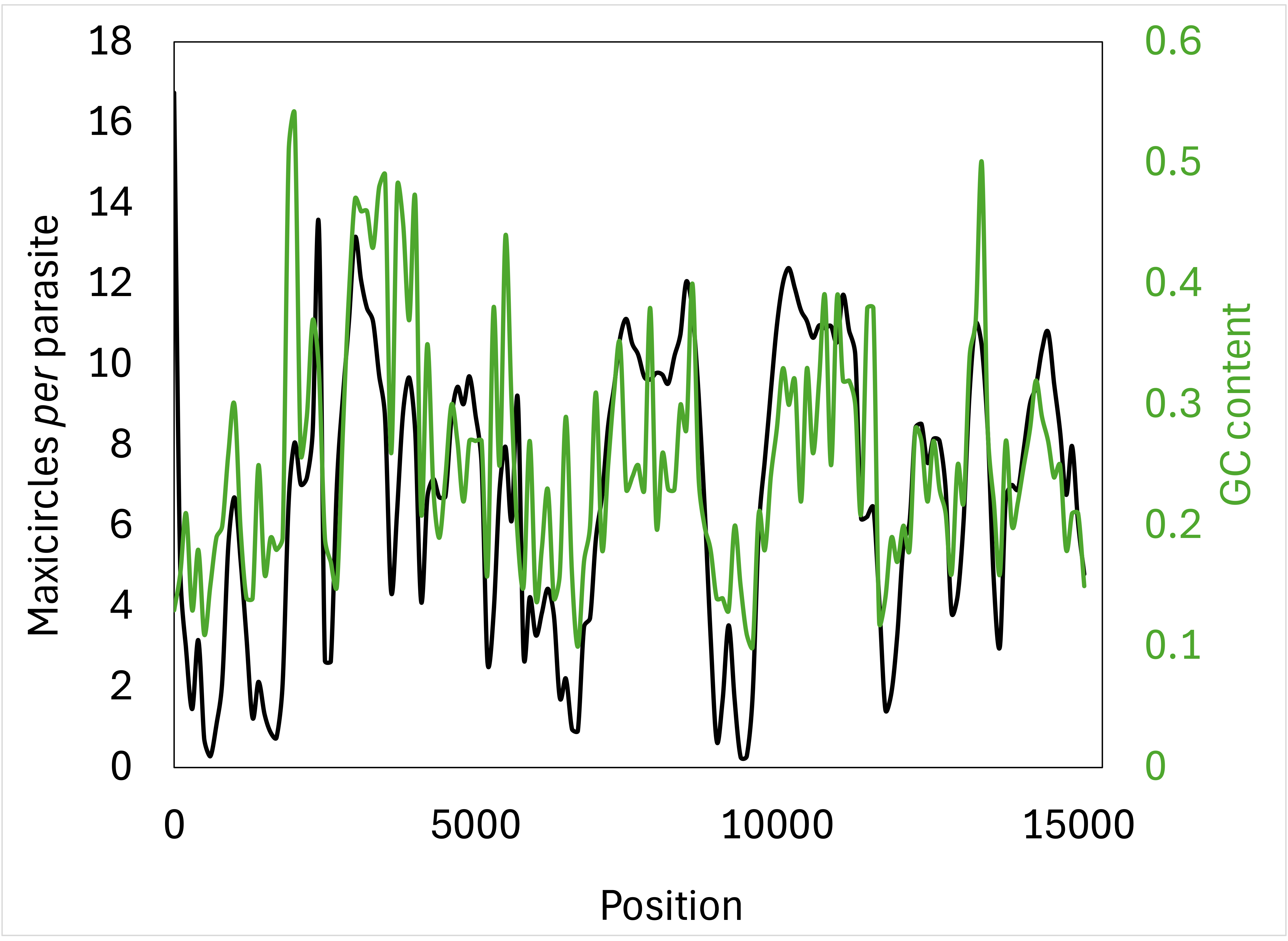

Supplement: Supplementary file 1 [file biology-14-01394-s001.zip › FigureS1.tif]
